# The effect of subacromial decompression on the curative effect of arthroscopic treatment of shoulder calcific tendinitis

**DOI:** 10.3389/fsurg.2022.1043794

**Published:** 2023-01-06

**Authors:** Feng Zhao, Jianbo Wu, Dong Wang, Peng Li, Wei Tian, Wenzheng Li, Bo Chai, Yuming Zhang

**Affiliations:** The Fifth Clinical Medical College of Shanxi Medical University, Taiyuan, China

**Keywords:** calcific tendinitis, rotator cuff, debridement, arthroscopic, subacromial decompression

## Abstract

**Objective:**

To observe and analyze the surgical efficacy of arthroscopic debridement of calcified deposits and arthroscopic debridement combined with subacromial decompression in patients with supraspinatus tendon calcific myositis. To observe the effect of Subacromial decompression on the efficacy of arthroscopic treatment of shoulder calcific tendinitis.

**Patients and methods:**

From 2016 to 2021, 48 cases of shoulder arthroscopic debridement due to supraspinatus calcific tendinitis met the inclusion criteria and were included, with 24 cases assigned to the arthroscopic debridement group and 24 cases to the arthroscopic debridement combined with subacromial decompression group. Changes between preoperative and postoperative shoulder pain and shoulder function were statistically analyzed.

**Results:**

The 24 patients in the arthroscopic debridement group were better than the arthroscopic debridement combined with subacromial decompression group in terms of short-term postoperative shoulder pain and shoulder joint function recovery (*P* < 0.05). There was no significant difference in the postoperative long-term shoulder pain and shoulder function recovery between the two groups (*P *> 0.05).

**Conclusions:**

Compared with arthroscopic debridement combined with subacromial decompression, arthroscopic debridement alone is a better surgical option for the treatment of calcific tendinitis.

## Introduction

Shoulder calcific tendinitis, which usually affects patients between the ages of 30 and 60 years and is more common in women than men, is a painful disease characterized by single or multiple calcium eposits within the rotator cuff (1). The deposits, composed of crystalline or amorphous hydroxyapatite (2), are most often located at the midsection or insertion of the supraspinatus tendon (3, 4). Uhthoff and Loehr described three clinical stages of the disease: precalcification, calcification, and postcalcification (6). In the early stages of calcification, fibrocartilaginous metaplasia and matrix vesicles combine to form calcified deposits. During the resting phase (calcification phase), the condition is dormant. When resorption finally occurs, fibroblasts and granulation tissue replace most of the calcified deposits (5, 6). The main clinical manifestation of calcific tendinitis of the shoulder joint is acute pain at onset, which may be accompanied by limitation of motion and muscle spasm (1). However, acute pain episodes with intermittent episodes of tendinopathy can also be observed in the chronic phase (7).

Rotator cuff calcific tendinitis is a recognized clinical disease of unknown etiology. Codman (8) first described the morphology and composition of calcified deposits and proposed that they typically occur within 1 cm proximal to the insertion of the greater tubercle of the humerus. The hypovascular area of the tendon has the worst blood supply and is most affected by stress. This area is often referred to as the “danger zone” because it is where tendon degeneration and necrosis are most likely to occur during the repair of tendon fiber ruptures. The local environment is acidic, which is conducive to the precipitation of amorphous free calcium ions and the formation of calcium salts. These calcium salts are deposited in tendon fibers, causing supraspinatus calcific tendinitis. McLaughlin (9) proposed that the earliest damage to myofibers is hyaline degeneration, followed by fibril formation and separation from the surrounding normal tissue. Continued wear results in the formation of necrotic fragments of the detached, crimped tendon. This is followed by the formation of atheroma, which leads to calcification. A nondegenerative, cell-mediated mechanism of calcification was subsequently proposed by Uthoff et al. (6) This mechanism induces metaplasia of normal tendons, which proceeds through cycles of formative and absorptive calcification. It is mediated by multinucleated giant cells and ultimately leads to a remodeling process whereby affected tendons reform into normal tendons. Several treatment options have been described for managing rotator cuff calcific tendinitis, but the consensus is that conservative management should be the first choice (3, 5). Nonsurgical interventions focus on reducing pain, including rest, non-steroidal anti-inflammatory drug (NSAIDs), steroid injections, and physical therapy. Minimally invasive techniques include ultrasound-guided lavage and extracorporeal shockwave therapy. Ultrasound-guided lavage and puncture aspiration can be performed under local anesthesia, and satisfactory results have been reported after 3 months. According to Hurt and Baker (10), in roughly 90% of patients, nonsurgical treatment provides relief. However, Wittenberg (11) and others have contended that surgical treatment can produce better long-term outcomes. Surgical treatment usually involves debridement of calcified deposits or debridement of calcified deposits with subacromial decompression. However, whether subacromial decompression with removal of calcified deposits improves patient outcomes remains unclear.

This retrospective study thus compared the outcomes of two surgical procedures for calcific tendinitis: arthroscopic debridement alone (Group A) and arthroscopic debridement with subacromial decompression (Group B). We hypothesized that arthroscopic debridement of calcified deposits alone would be equal, in terms of efficacy, to arthroscopic debridement combined with subacromial decompression in patients with calcific tendinitis of the supraspinatus tendon.

## Patients and methods

This retrospective study included 48 patients with symptomatic supraspinatus tendon calcification who underwent surgery in Shanxi Provincial People's Hospital from 2016 to 2021. All methods were carried out in accordance with relevant guidelines and regulations. All experimental procedures followed the Declaration of Helsinki.

Patients were included if they were between the ages of 18 and 60 years and exhibited isolated calcified deposits in the supraspinatus tendon with an intact rotator cuff before surgery. Patients received one or more of the following interventions: oral antibiotics, anti-inflammatory medications, subacromial steroid injections, supervised physical therapy, and a self-directed exercise program. We followed patients for at least 6 months.

Patients with other shoulder pathologies, such as biceps longus pathology, shoulder instability, significant acromion impingement, acromioclavicular osteoarthritis, or dystrophic calcification, were excluded. Patients were excluded if their calcified deposits were found on radiographs or magnetic resonance imaging or during surgery. Patients were also excluded if they had undergone previous shoulder surgery or trauma or had experienced symptoms for more than 12 months.

### Surgical procedures

**Figure 1:**
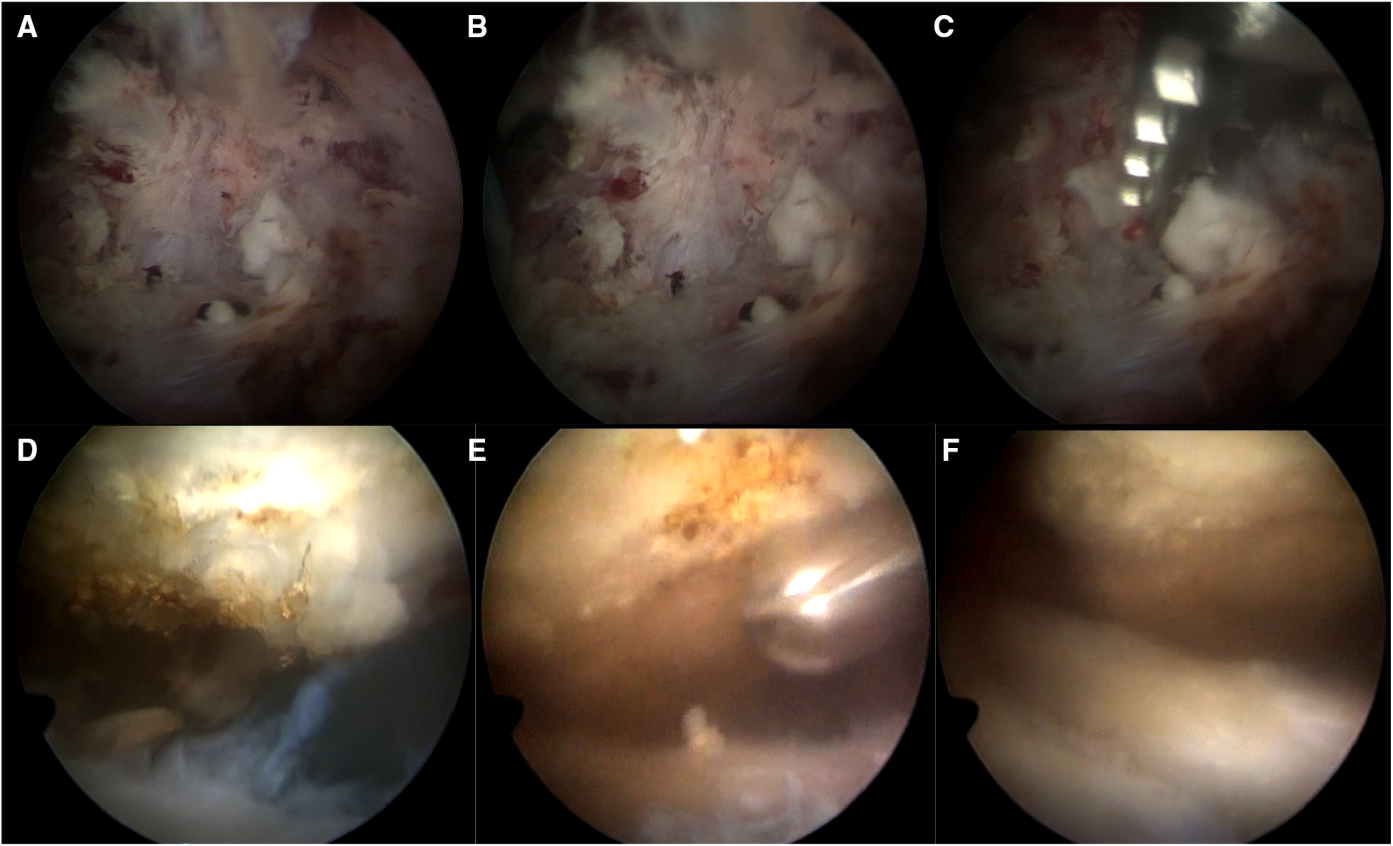
Intraoperative images: (**A**) Group A clean the synovium in the joint cavity, and toothpaste-like white calcification spillage can be seen. (**B**) Group A stripped the calcified surface capsule to promote the flow of toothpaste-like white calcification. (**C**) Group A used soft tissue grippers and planers to debridement around calcifications. (**D**) Group B images before subacromial decompression. (**E**) Group B used a planer to debridement the acromion. (**F**) Picture of the surgical area after subacromial decompression in Group B.

All operations were performed by the same senior surgeon. Surgeons in Group A performed arthroscopic debridement of calcified deposits only. Surgeons in Group B performed debridement in the same manner but with subacromial decompression. All patients in both groups were under general anesthesia. After the anesthesia was administered, the patients were placed in the lateral decubitus position. The affected shoulder was suspended and fixed with traction of the clavicle and acromion. The coracoid process was marked, and routine disinfection and draping were performed. A standard posterior approach was adopted, and an observation channel was established. The surgeon first examined whether the glenoid joint was intact, the long head of the biceps tendon was intact, and the hyperplasia in the shoulder joint was severe. Subsequently, a standard anterior approach was established, and the glenohumeral joint and subacromial space were examined. Microscopically, the surgeon looked for whether synovial tissue in the joint cavity exhibited congestion and edema and whether the joint capsule exhibited an inflammatory reaction. The subacromial bursa and the synovial membrane of the joint capsule were examined for villi-like adhesions and dot-like white calcified deposits. When clearing the subacromial bursa space, the surgeon examined whether the joint capsule showed the “strawberry sign” indicating local congestion and edema. The surgeon then made an incision along the direction of the supraspinatus tendon and longitudinally cut the surface capsule of the calcification, prompting the flow of toothpaste-like white calcifications, with high tension. The surgeon used soft-tissue-grasping forceps, nucleus pulposus forceps, and a planing knife to fully debride the surrounding calcifications. The assistant rotated and squeezed the shoulder joint to remove the calcification to the maximum extent possible. Finally, the tension and continuity of the supraspinatus tendon were examined. The surgeons performed subacromial decompression on patients in Group B (Figure 1).

On the first postoperative day, passive shoulder exercise (pendulum movement) was started, and the abduction and flexion angle of the shoulder joint was controlled within 90°. The passive forward flexion and abduction of the shoulder joint gradually reached the normal range, and x-rays were reviewed. At 3 weeks after the operation, the active exercise of the shoulder joint helped the shoulder joint to be able to abduct more than 90°. To strengthen the shoulder joint, exercises included climbing the wall with fingers and other active activities. In the third week after the operation, we focused on exercising the rotator cuff muscles and deltoid muscles.

### Evaluation indicators

All patients were followed up for at least 6 months after the operation and underwent outpatient reexamination. The patients were regularly reexamined in the orthopaedic outpatient clinic at 1 week, 1 month, 3 months, and 6 months after the operation. The VAS pain score was used to evaluate the shoulder joint pain at each follow-up. At 1 month, 3 months, and 6 months after the operation, the Constant–Murley score was used to evaluate the shoulder joint function before and after treatment, and the postoperative rehabilitation exercise plan was adjusted according to the shoulder joint function.

### Statistical analysis

We used *t* test or ×2 test to compare the age distribution, preoperative shoulder VAS score, and Constance-Murray score of the two groups to determine whether the preoperative data were comparable. After that, the independent sample paired t test was used to analyze the VAS pain and Constant–Murley score of the two groups of patients before and after surgery to determine whether there was statistical significance. All analyses were performed with SPSS 28.0 statistical software for statistical analysis of the sorted data.

## Results

From 2016 to 2021, 48 patients with shoulder pain and calcific tendinitis were treated. Group A included 10 male patients and 14 female patients who were aged 40 to 68 years (49.93 ± 6.773 years). The preoperative VAS score (Visual analogue scale) in group A was (7.85 ± 1.37) points, and the Constant–Murley score was (45.54 ± 12.53) points. Group B included 11 male patients and 13 female patients aged 46 to 67 years (52.14 ± 7.211 years). The preoperative VAS score in Group B was 7.54 ± 1.42 points, and the Constant–Murley score was 46.17 ± 11.54 points.

**Figure 2:**
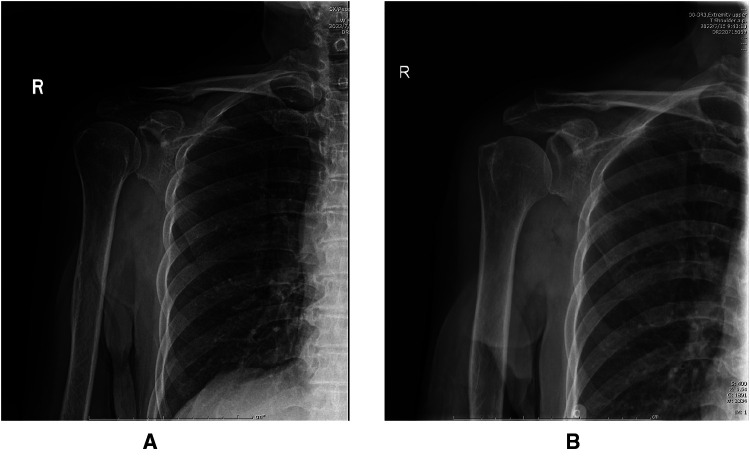
(**A**) Preoperative x-ray of the patient with supraspinatus calcific tendonitis calcifications. (**B**) 1 week after surgery, calcifications cannot be detected on x-rays.

**Figure 3:**
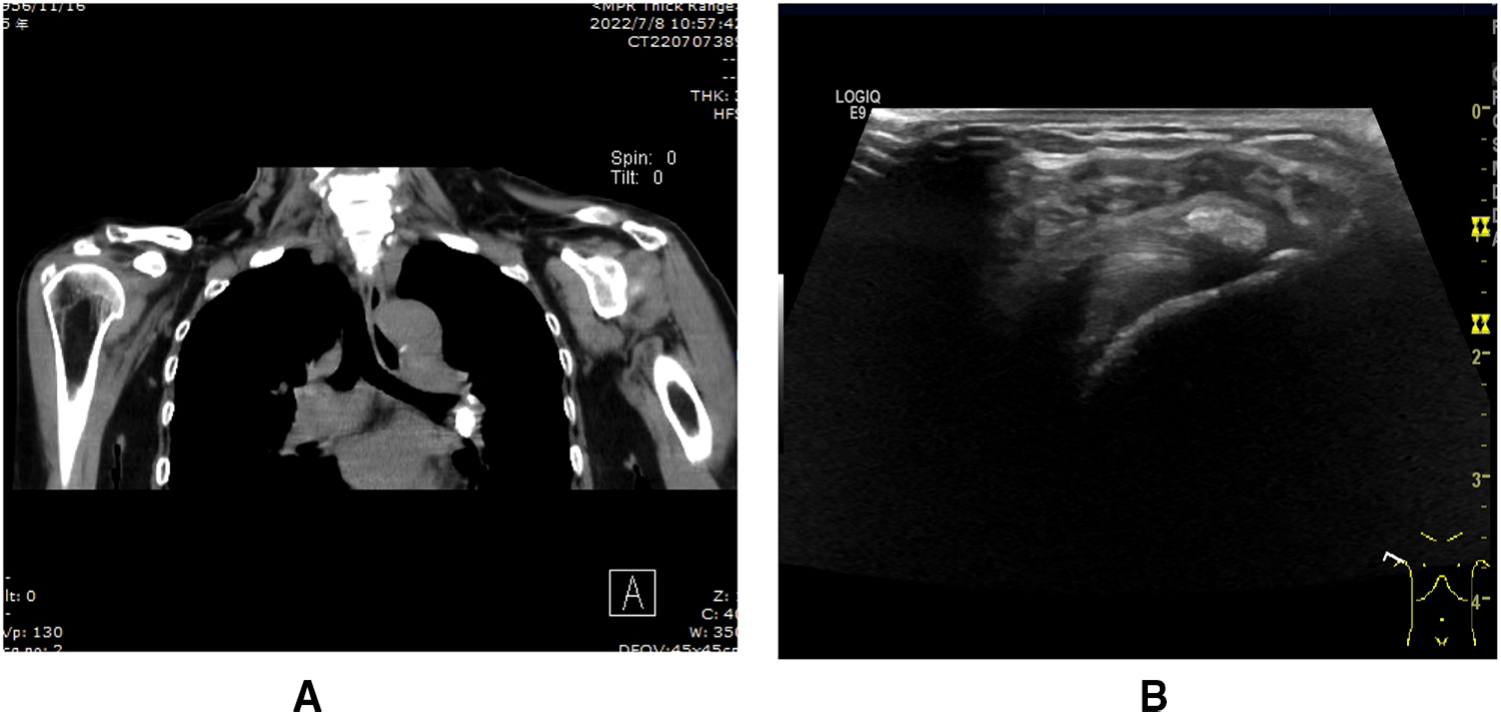
(**A**) Calcification foci of supraspinatus tendonitis on preoperative CT. (**B**) Calcific tendonitis calcification foci on preoperative shoulder. Ultrasound.

**Figure 4:**
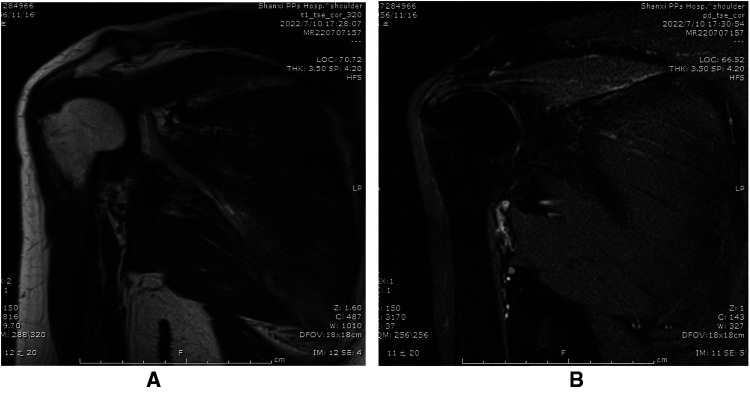
Preoperative MRI images: (**A**) calcified foci of supraspinatus tendonitis in preoperative T1WI. (**B**) Calcific tendonitis calcifications in preoperative T2WI.

During the follow-up period, all patients' symptoms improved significantly (Figures 2–4). No significant difference existed in age distribution between the two groups (*P* = 0.532). In terms of shoulder joint function, 1 month after the operation, the difference in Constant–Murley scores of Group A (65.50 ± 10.65) and Group B (54.23 ± 11.35) was statistically significant (*P* < 0.01). However, no significant difference existed between the two groups at 3 months and 6 months after the operation. Significant differences existed in postoperative VAS scores between the two groups at 1 week and 1 month after operation (*P* < 0.05), but with no significant difference at 3 months and 6 months after operation. See Tables 1, 2.

**Table 1:** Comparison of Constant–Murley scores at each follow-up point in the two groups.

Group	Number of cases	Age	Constant–Murley score			
			Preoperative	1 month	3 month	6 month
Group A	24	49.93 ± 6.733	45.54 ± 12.53	65.50 ± 10.65	78.50 ± 11.50	86.50 ± 13.24
Group B	24	52.14 ± 7.211	46.17 ± 11.54	54.23 ± 11.35	75.43 ± 10.75	84.50 ± 12.32
*t* value			−0.17	2.79	1.78	0.44
*P* value		0.532	>0.05	<0.01	>0.05	>0.05

**Table 2:** Comparison of VAS scores at each follow-up point in the two groups.

Group	Number of cases	VAS score				
		Preoperative	1 week	1 month	3 month	6 month
Group A	24	7.85 ± 1.37	2.83 ± 1.21	1.93 ± 1.28	1.28 ± 1.19	1.01 ± 1.34
Group B	24	7.54 ± 1.42	5.18 ± 1.38	3.15 ± 1.23	1.52 ± 1.10	1.10 ± 1.20
*t* value		0.67	−5.69	−2.73	−0.78	−0.23
*P* value		>0.05	<0.001	<0.05	>0.05	>0.05

## Discussion

The calcific tendonitis of the shoulder joint is most common with calcification of the supraspinatus tendon, and the incidence in women is higher than that in men (1, 12, 13). The etiology of calcific supraspinatus tendonitis is not fully understood. Local pain in the shoulder joint is the main manifestation in patients with early calcific supraspinatus tendinitis, with or without shoulder joint movement limitation. In the advanced stage, the pain of the shoulder joint is gradually aggravated, and the pain is more obvious at night. It is easy to radiate to the neck and back and it is often combined with the limitation of shoulder joint movement. Some patients have severe pain during an acute exacerbation and severe pain at rest. Their pain becomes worse after the activity, which makes people afraid to actively move the shoulder joint. Eventually patients develop shoulder adhesions and eventually lose range of motion in the shoulder joint, which seriously affects their quality of life.

Conservative treatment can be used in the early stage of the disease, such as oral drugs, irrigation, mashing, partial closure, etc. And 90% of patients can be cured by conservative treatment. Some qualified hospitals can provide ultrasound or physical rehabilitation treatment, and most patients can obtain satisfactory results after a series of standardized non-surgical treatments (14). However, in some patients, conservative treatment is not effective, and surgery can be used at this time. Surgical treatment is divided into incision cleaning and arthroscopic treatment. Open surgery is gradually not accepted by patients and doctors due to its large trauma and slow postoperative recovery. The advantages of arthroscopic surgery for calcific supraspinatus tendinitis are that it is minimally invasive and less bleeding. It can avoid the deltoid muscle damage caused by open surgery. It can also avoid damage to the deltoid muscles during open surgery and reduce postoperative re-adhesions. Because of the rapid recovery of shoulder function after surgery, the arthroscopic surgery has gradually become the best choice for the treatment of this disease.

At present, there were two main views on the cleaning of calcifications in the shoulder joint. Some scholars believed that complete removal of the calcification of rotator cuff calcific tendinitis could ensure the effect of minimally invasive arthroscopic surgery for rotator cuff calcific tendinitis and adequate relief of postoperative shoulder pain (15, 16). Other scholars believed that good curative effect could be obtained by partially clearing the calcifications. Completely clearing the calcifications in the shoulder joint would result in damage to the normal tendon tissue in the shoulder joint. It did not affect the postoperative recovery effect (17, 18).

There was still a big controversy about whether or not acromoplasty was needed during the operation. Balke (19) advocated routine prophylactic acromoplasty for all patients. They believed that this would widen the subacromial space and avoid pain caused by subacromial impingement during postoperative shoulder movement. Marder (20) believed that whether or not acromoplasty was performed at the same time had no significant effect on the functional prognosis of the shoulder joint. Marder believed that simply cleaning up the calcified foci could reduce the surgical steps, shorten anesthesia and shorten the operation time. Finally, the incidence of complications was reduced.

This study compared and analyzed the therapeutic effects of arthroscopic debridement alone and arthroscopic debridement combined with subacromial decompression. The results demonstrated that arthroscopic debridement alone and arthroscopic debridement combined with subacromial decompression, in terms of VAS scores and Constant–Murley scores of the patients, both achieved significant improvement compared with their condition before surgery. However, the VAS scores of patients in Group A and Group B were significantly different at 1 week and 1 month after surgery. Compared with those in Group A, patients in Group B had slower recovery of shoulder joint function after the operation. No significant difference existed in VAS score or Constant–Murley score between Group A and Group B at 3 months and 6 months after the operation.

In general, arthroscopic calcification debridement has the advantages of less overall trauma, faster postoperative recovery, less deltoid muscle damage. And it is conducive to early postoperative rehabilitation exercises. It has the advantages of clear field and real-time detection of subacromial space lesions and timely treatment. There is no residual calcification after simple arthroscopy calcification foci cleaning compared with subacromial decompression, and there are no serious complications such as deltoid muscle injury and vascular injury. Most importantly, the overall satisfaction of the patients is high.

## Conclusion

This study found that compared with arthroscopic debridement alone, the simultaneous use of subacromial decompression delayed the time to return to normal motion of the shoulder joint, with no significant improvement in long-term outcomes and prolonged operation time. Patients spend more money on surgery. Therefore, we believe that subacromial decompression has no benefit as an additional procedure to remove calcified deposits in patients without significant acromial impingement. We believe that pure debridement of calcified deposits is a better surgical option for the treatment of calcific tendinitis.

## Limitation

This study had several limitations, and most importantly the location/size of the calcifc deposit and the severity of symptoms was diferent for each case, and this may have infuenced clinical outcomes in a manner that was difcult to control. This study was a short-term retrospective study with a short follow-up period, and the long-term efficacy of the surgery remains to be confirmed. Secondly, the number of cases in this study was small, the pathogenesis of calcific tendinitis was not clear, and a larger number of cases would help to understand the prognosis more accurately.

## Data Availability

The original contributions presented in the study are included in the article/Supplementary Material, further inquiries can be directed to the corresponding author.
